# Supplementary figures and images for: Effects of visual inputs on neural dynamics for coding of location and running speed in medial entorhinal cortex
Source: eLife. 2020 Dec 10;9:e62500. doi: 10.7554/eLife.62500 (PMC7773338; doi:10.7554/eLife.62500)

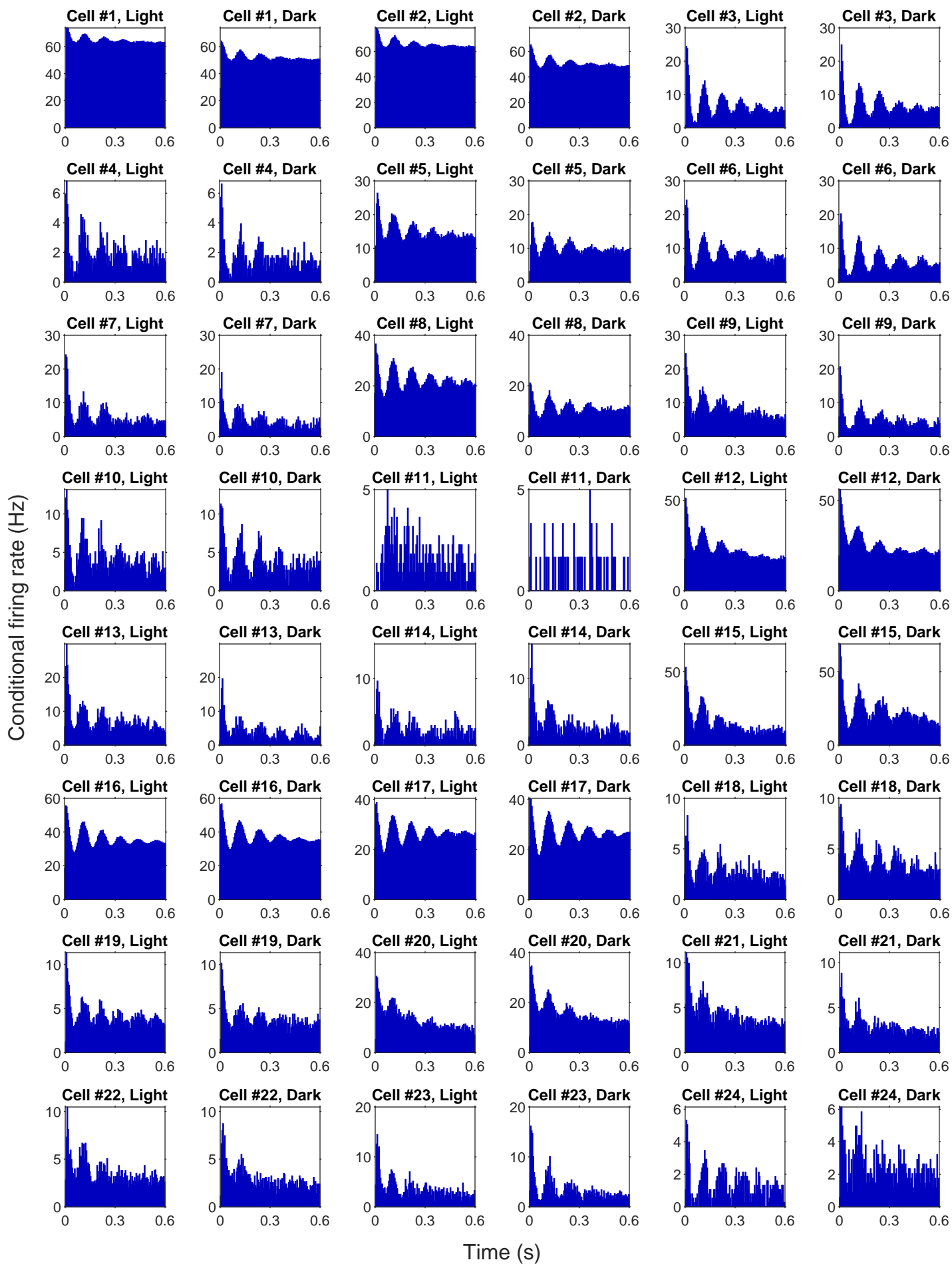

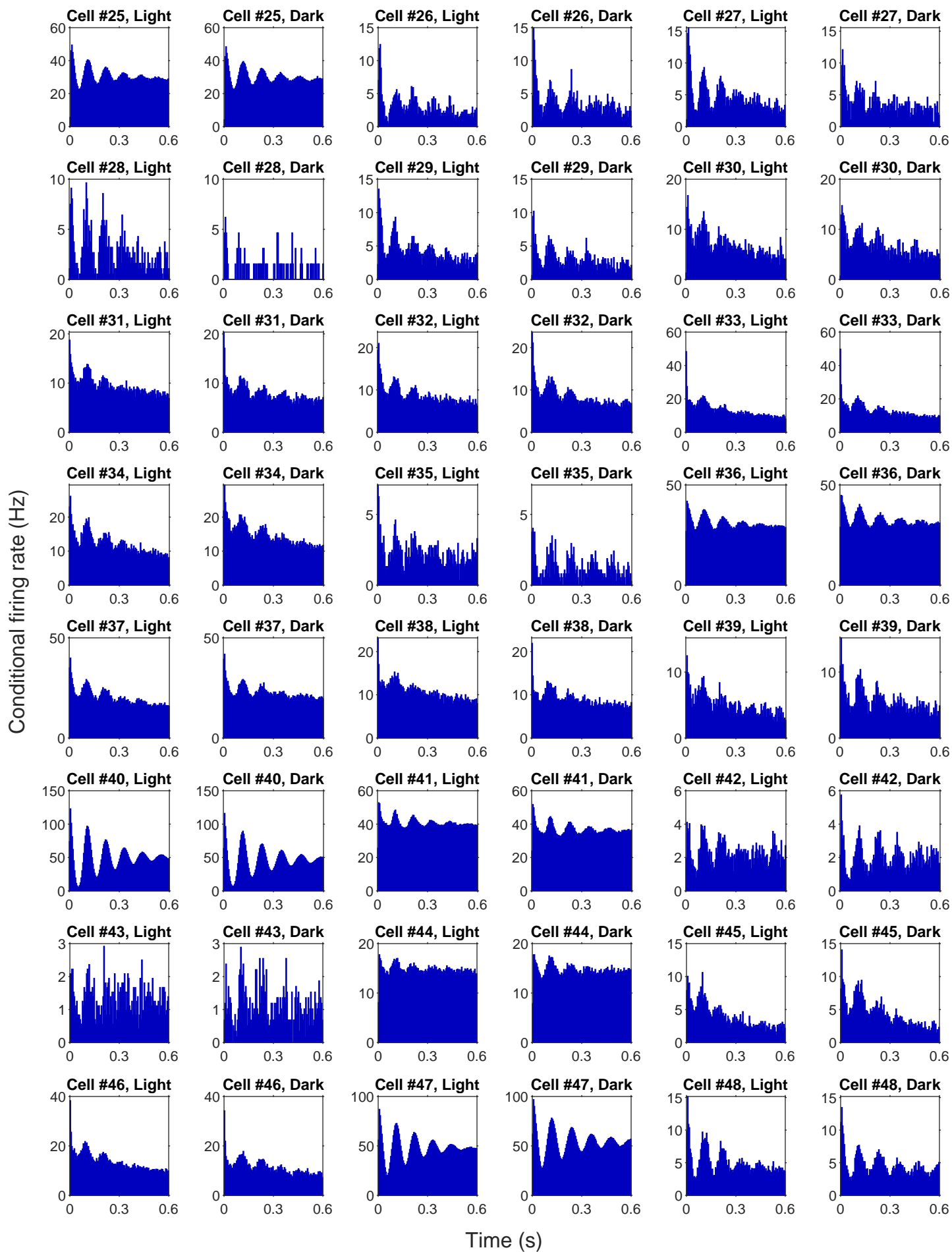

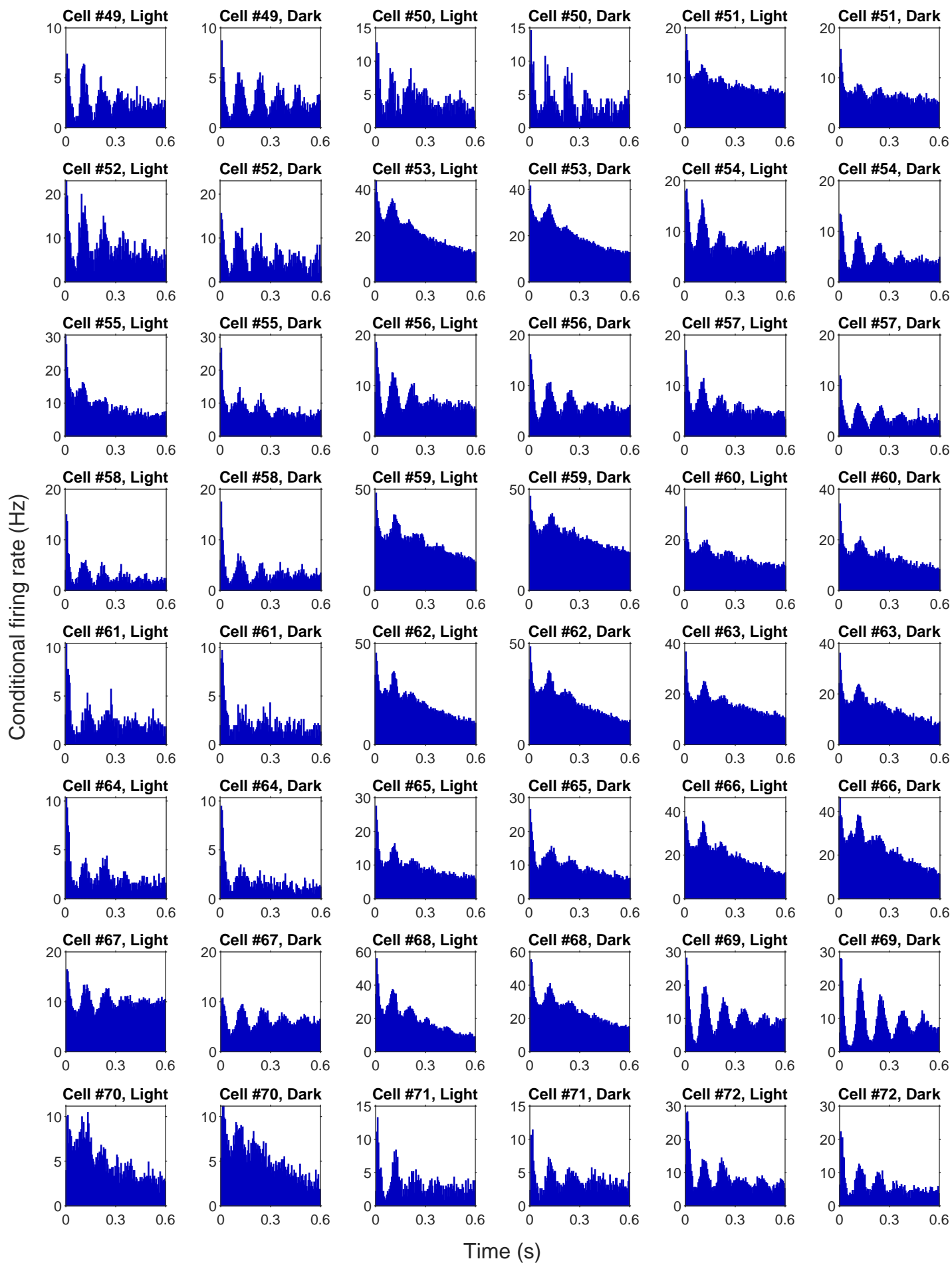

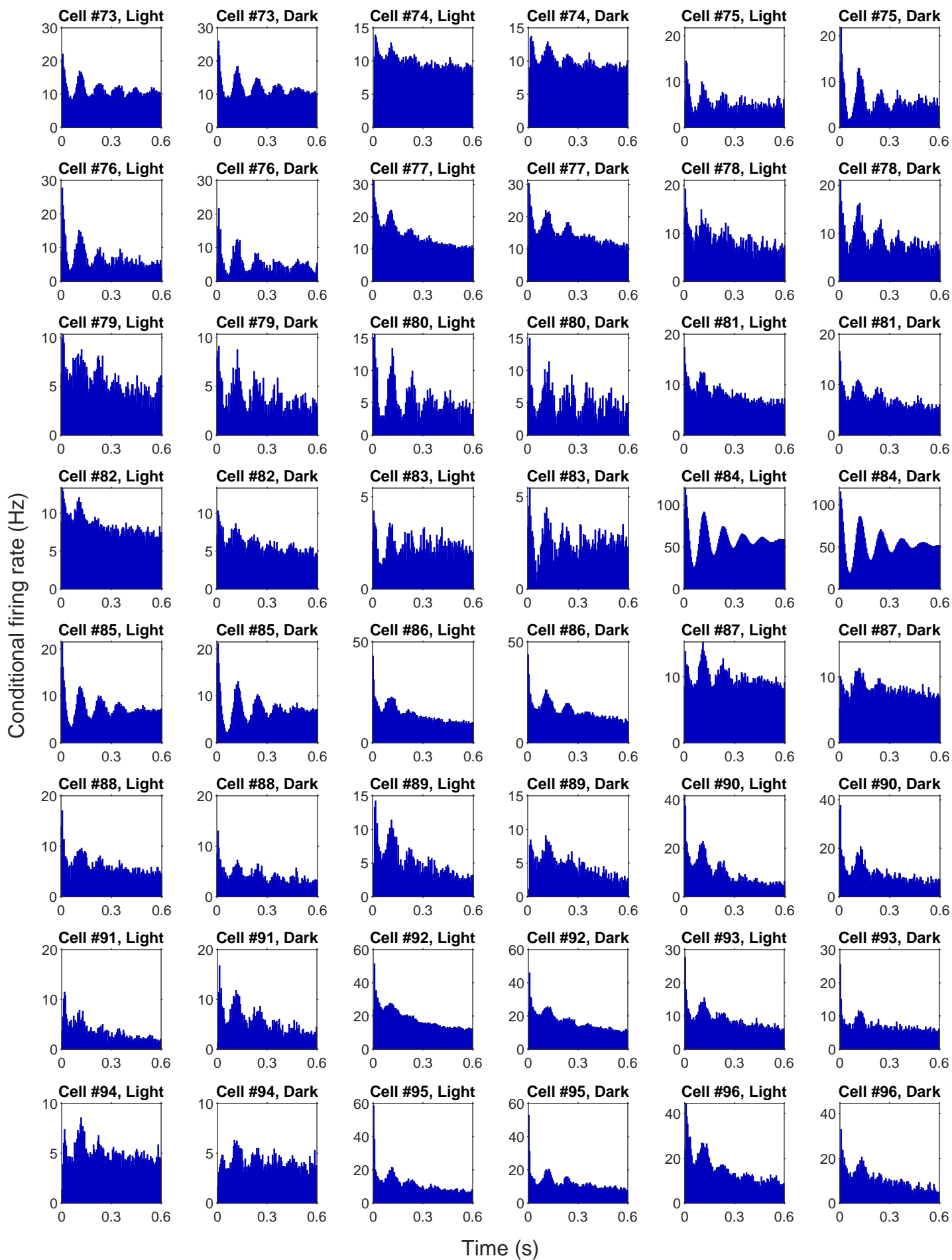

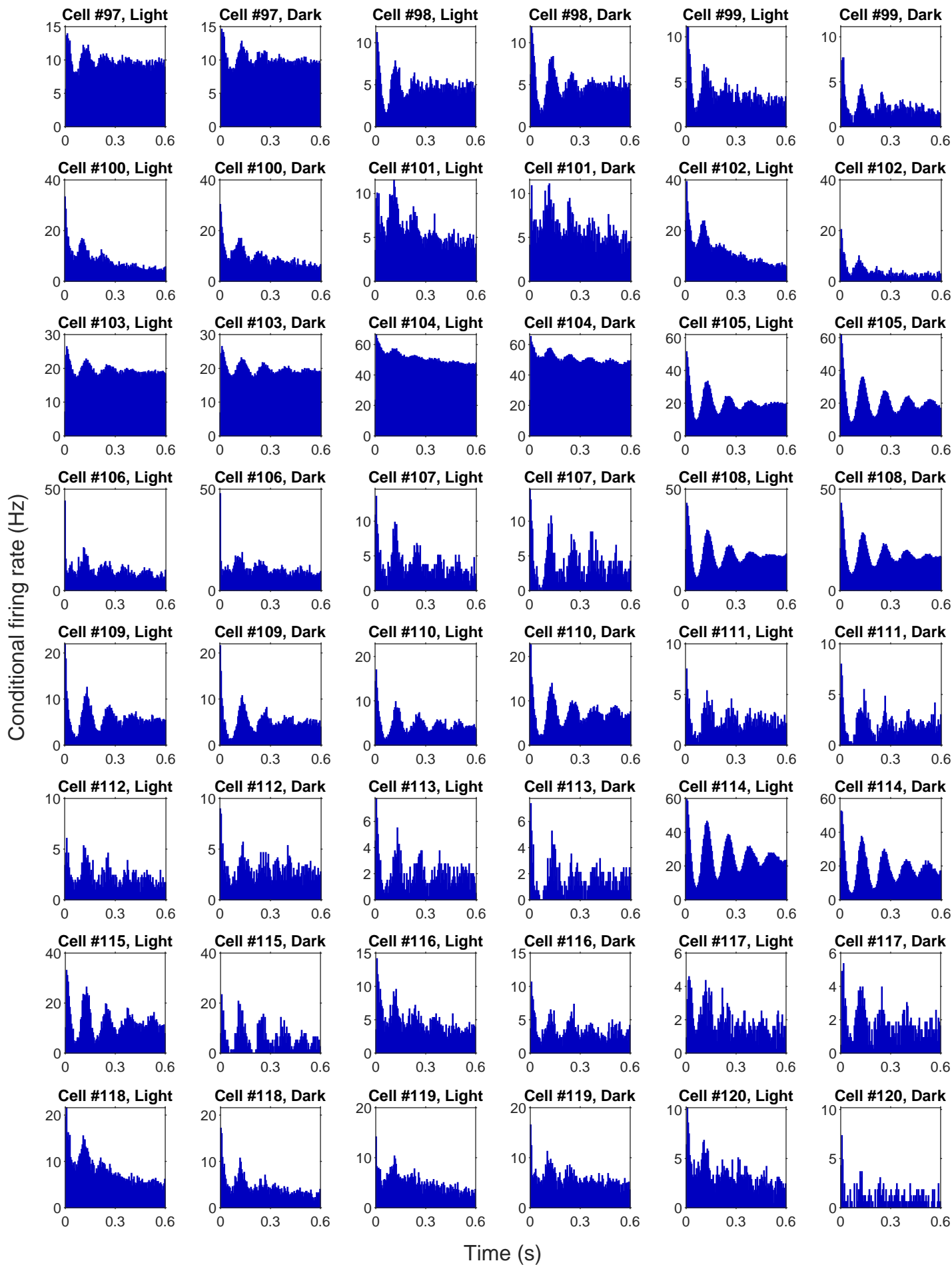

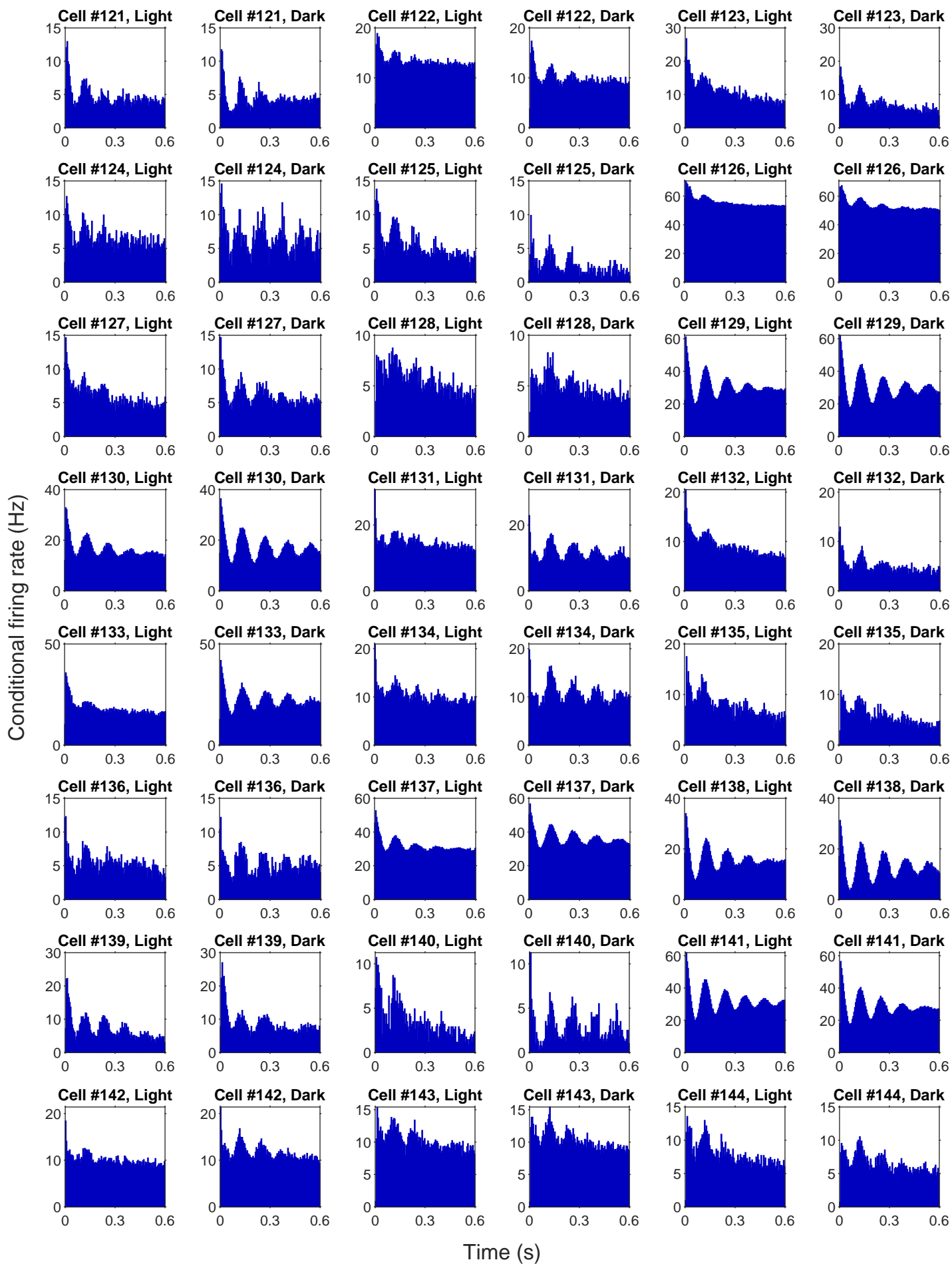

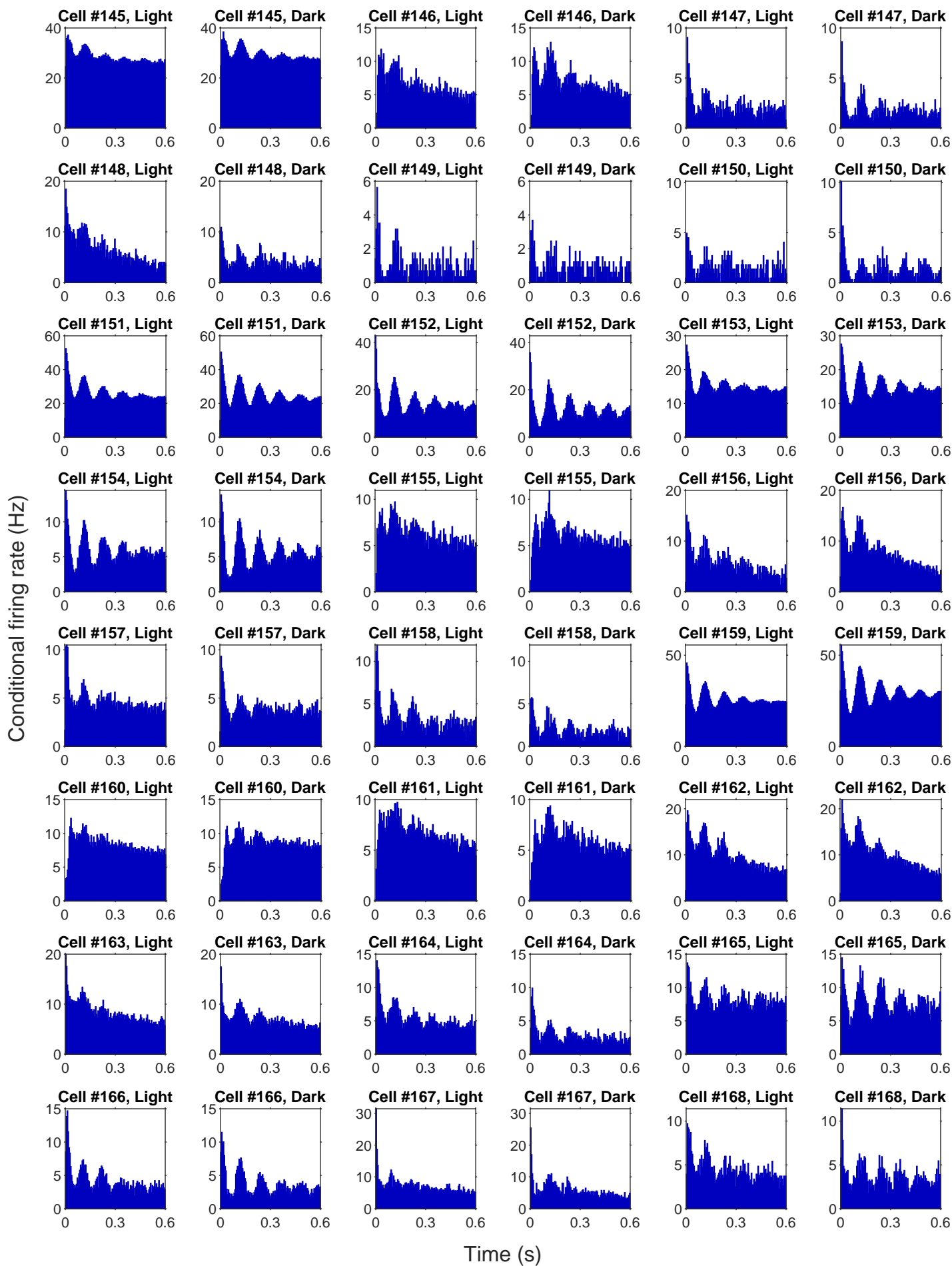

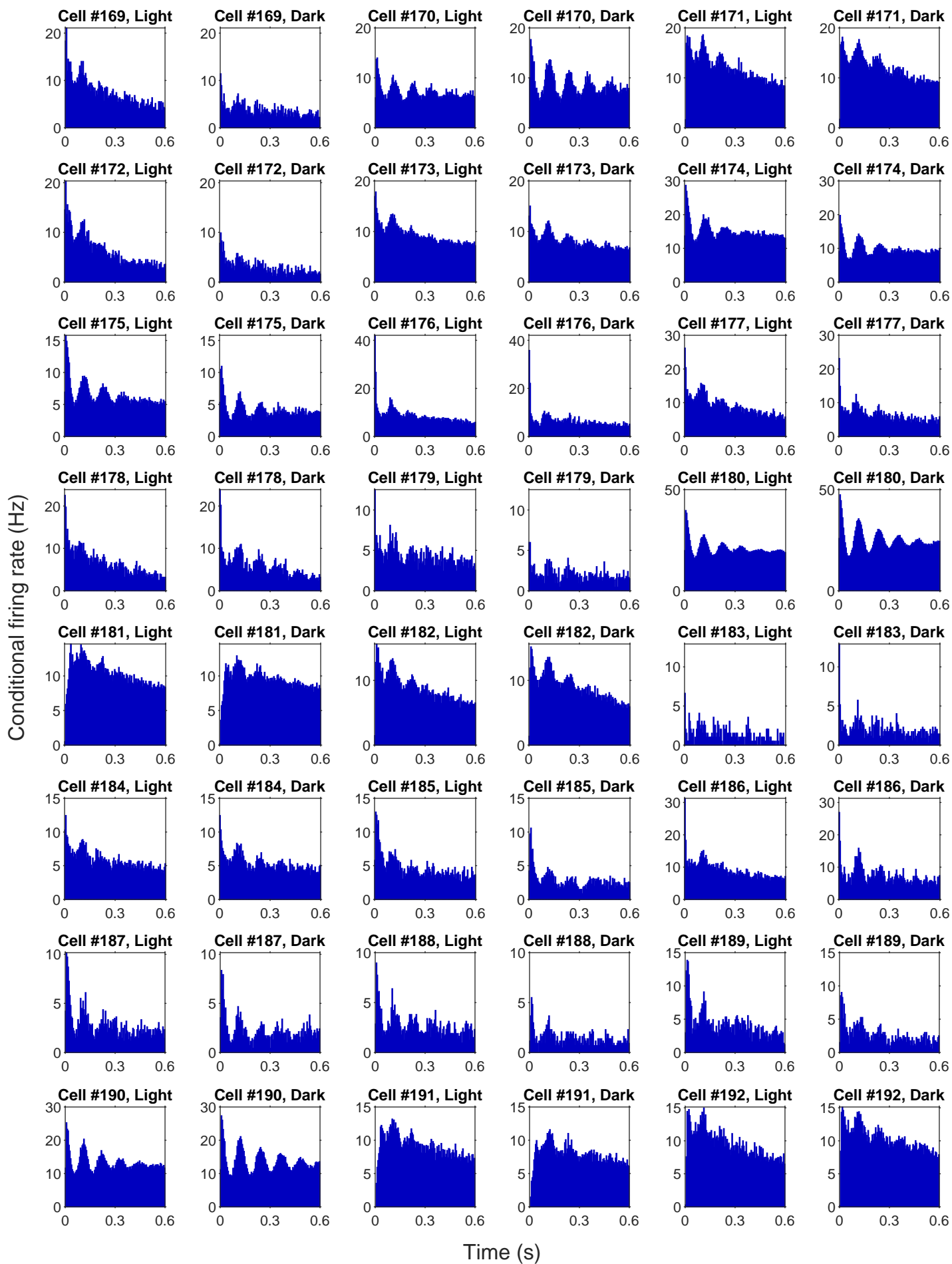

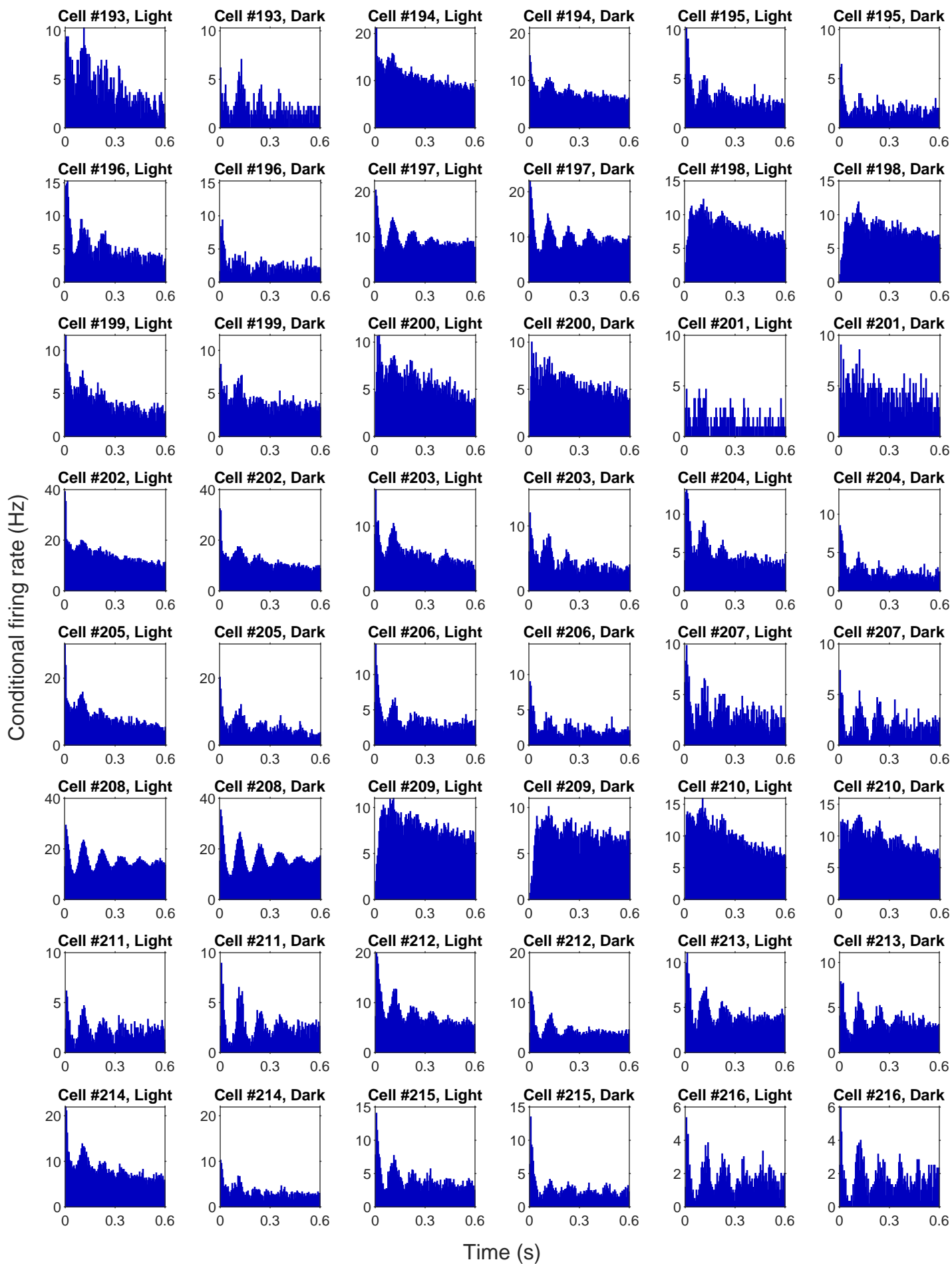

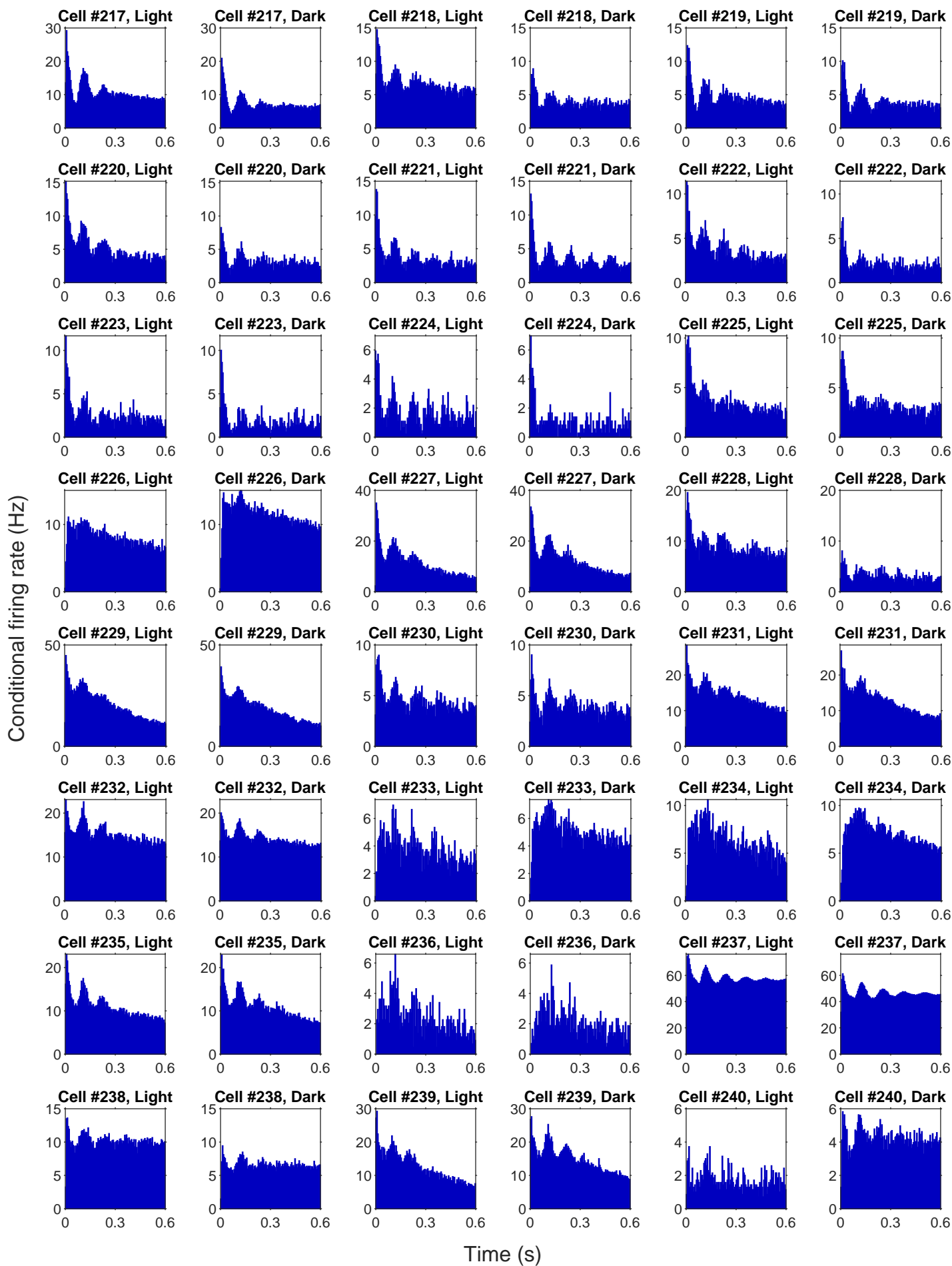

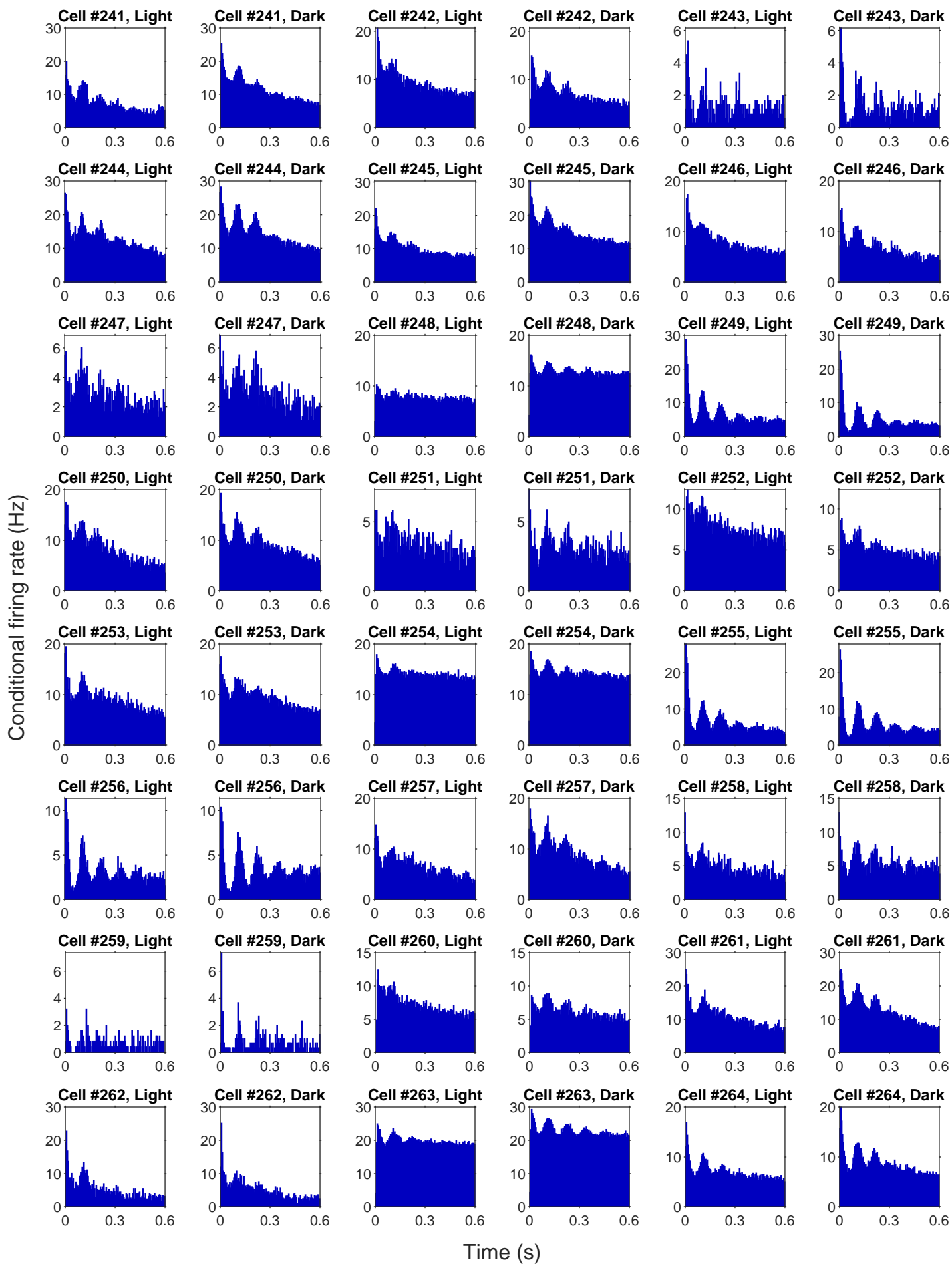

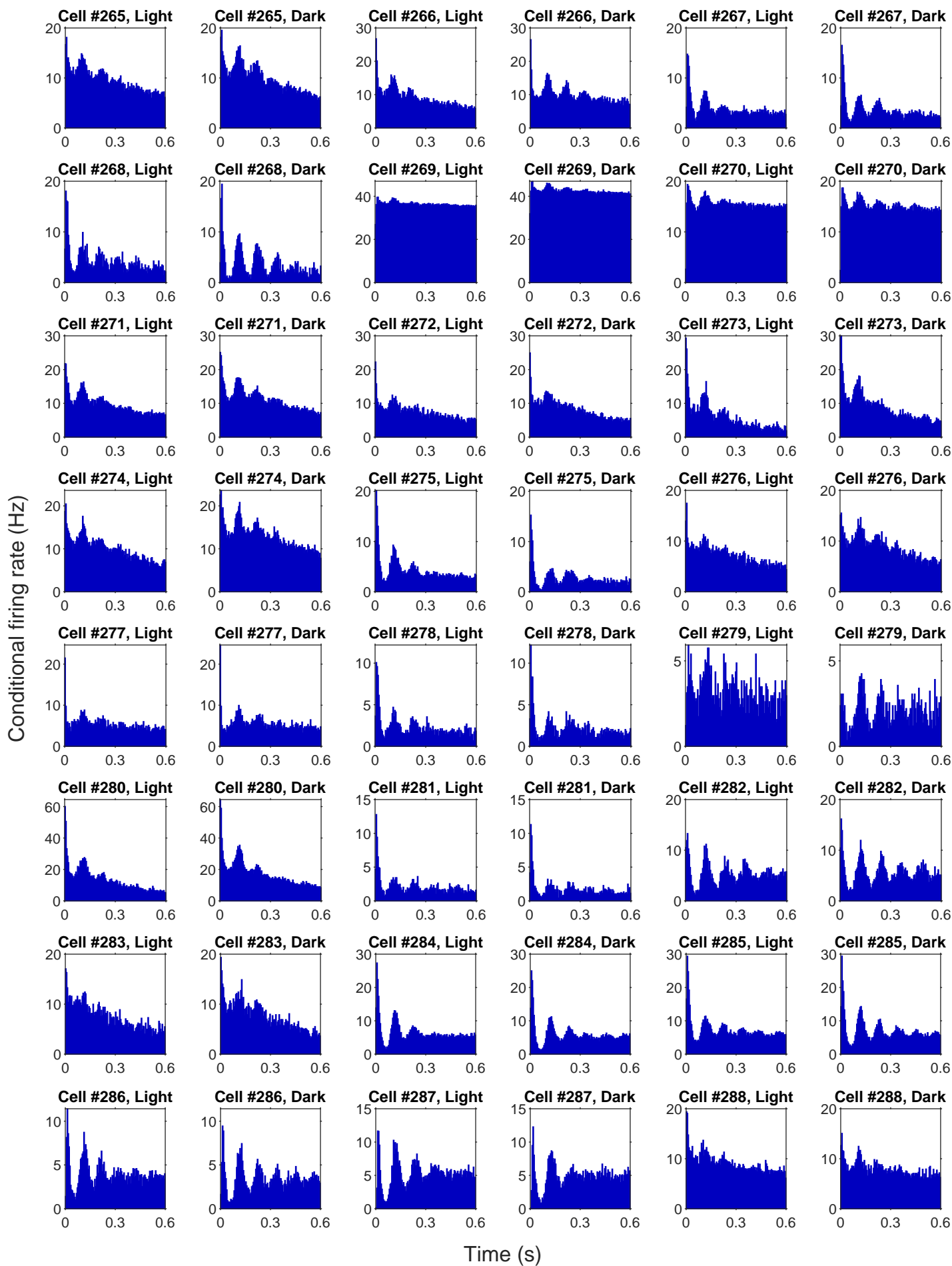

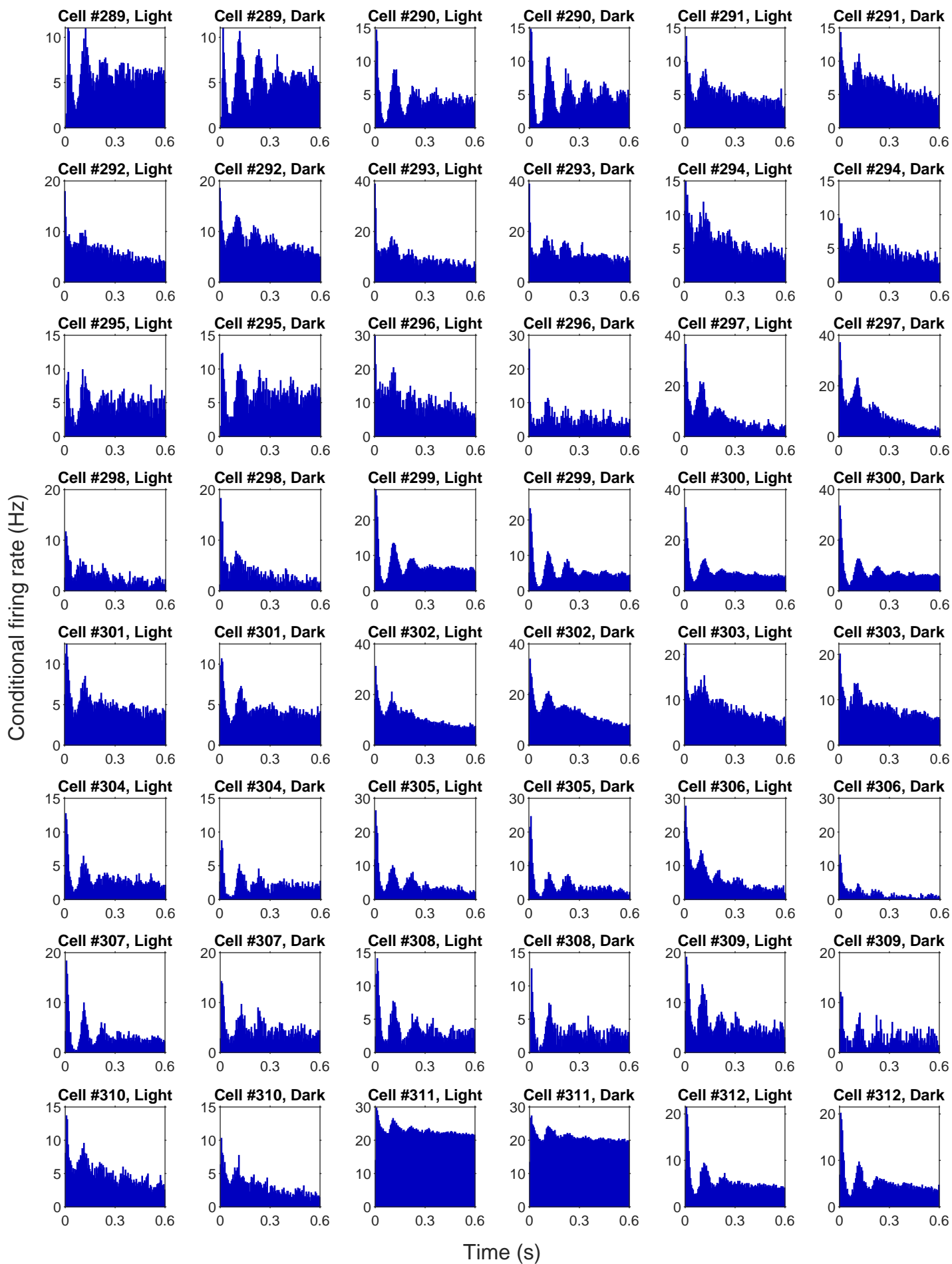

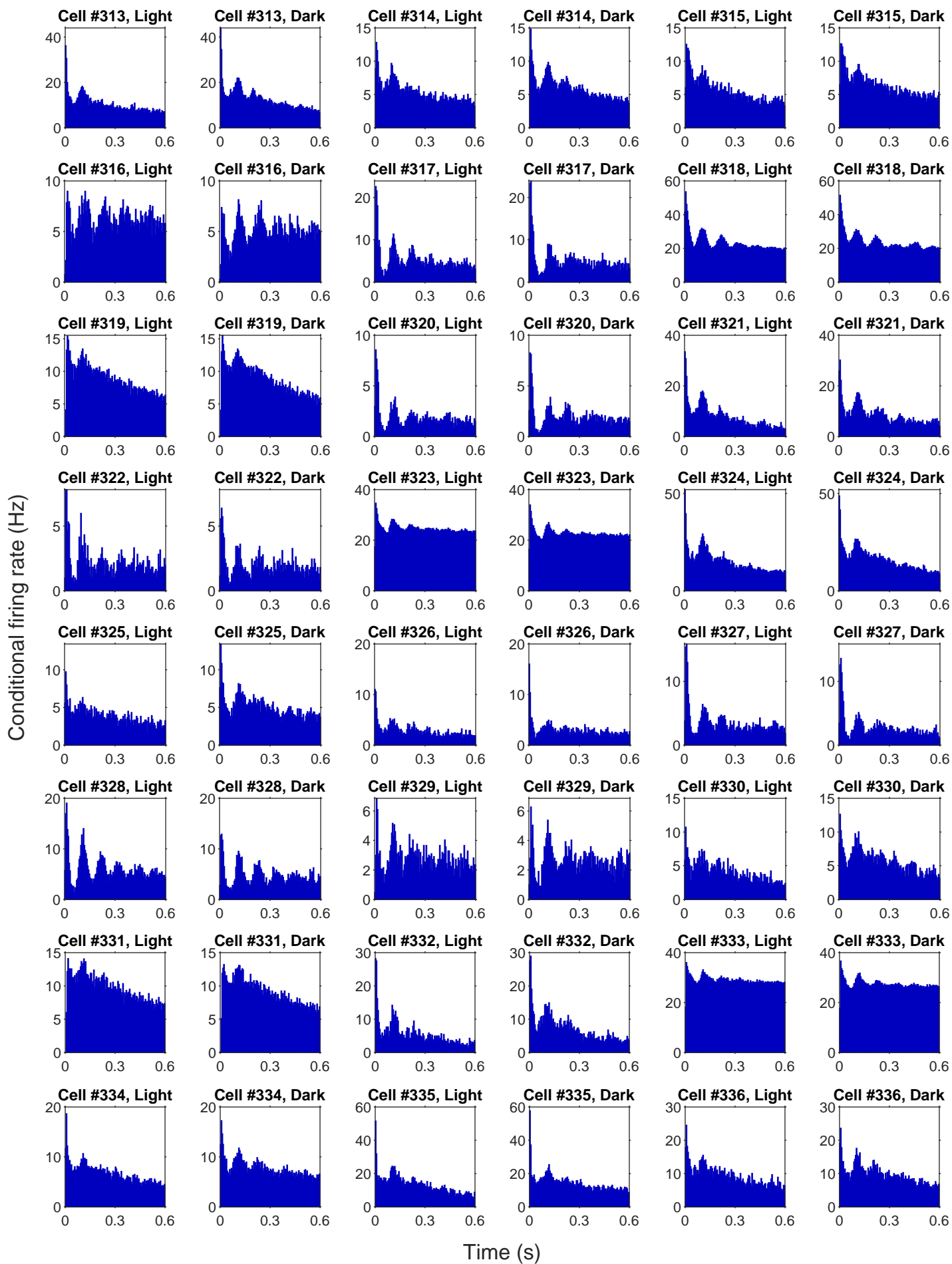

Conditional firing rate (Hz)

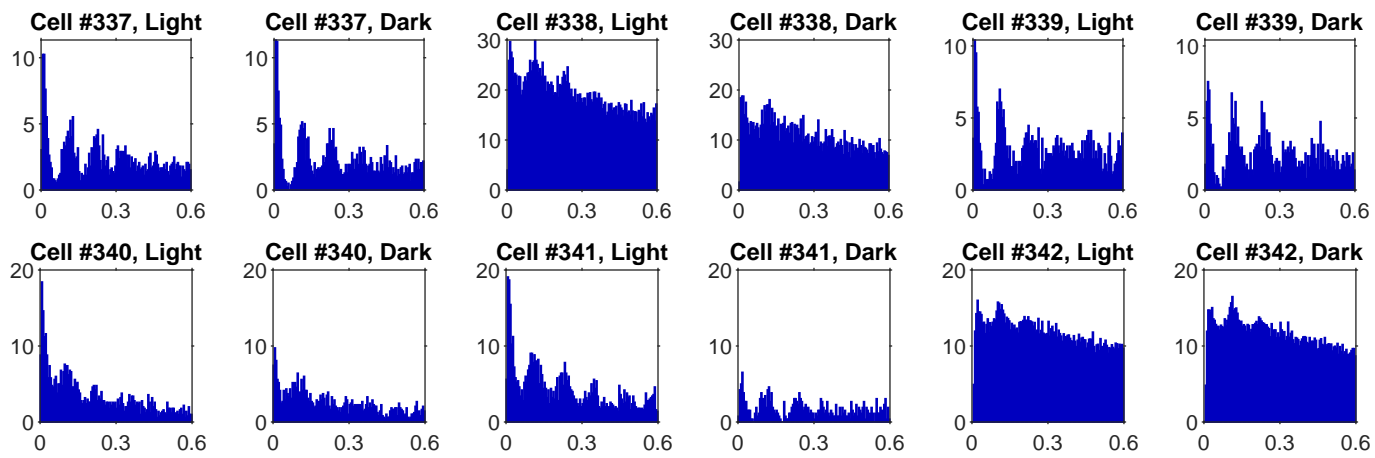

Time (s)

Supplement: Figure 2—source data 1. [file elife-62500-fig2-data1.pdf]

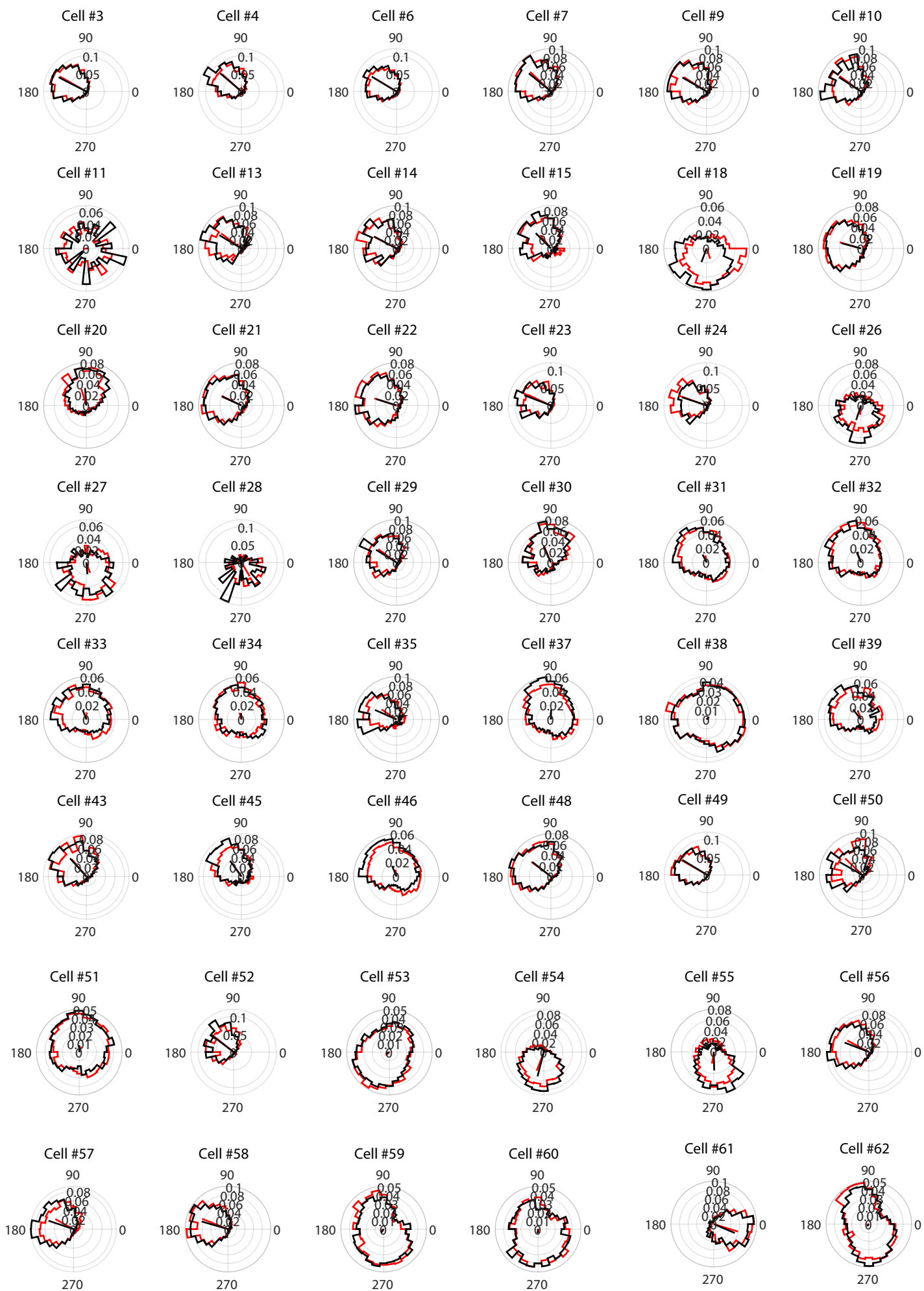

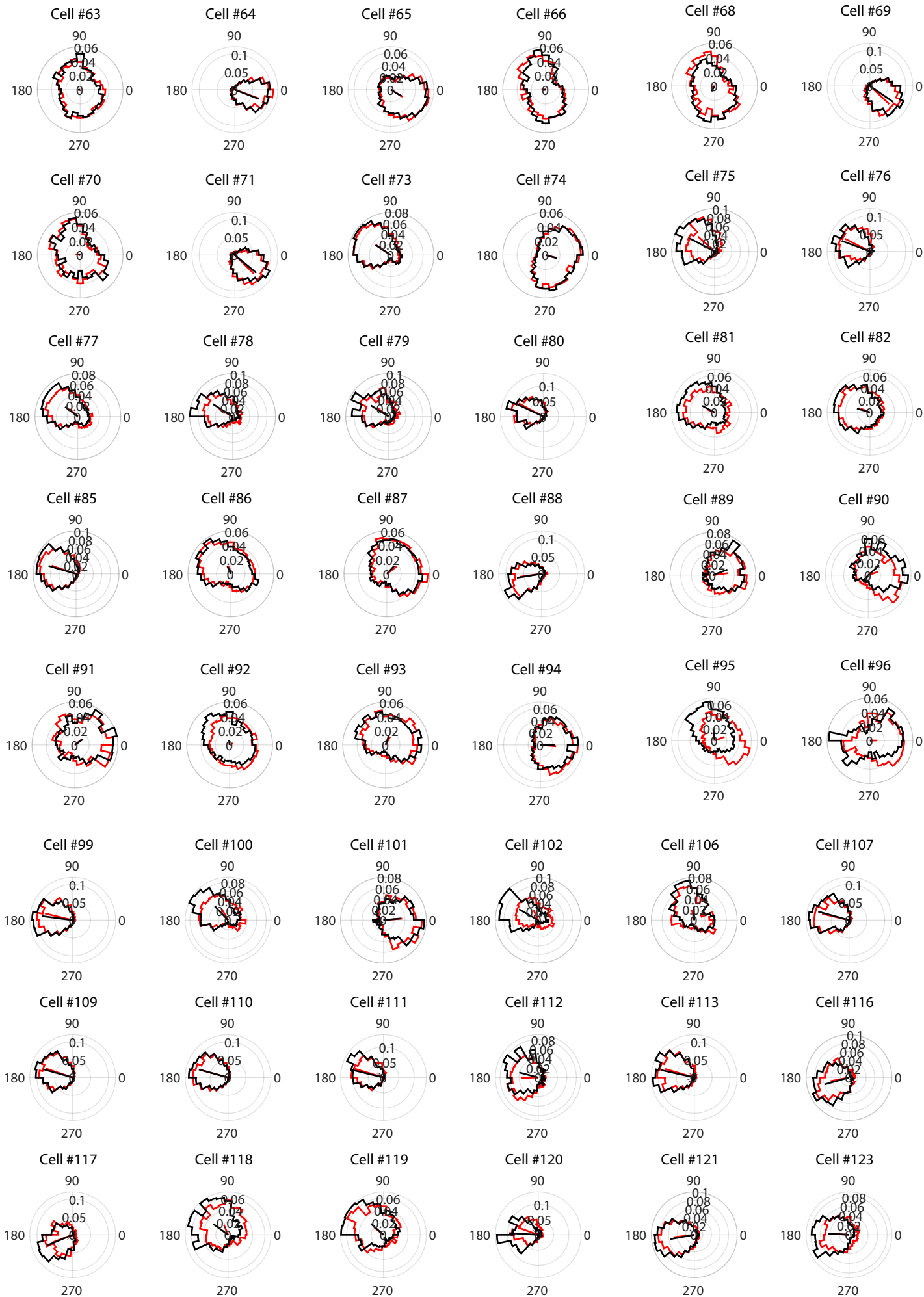

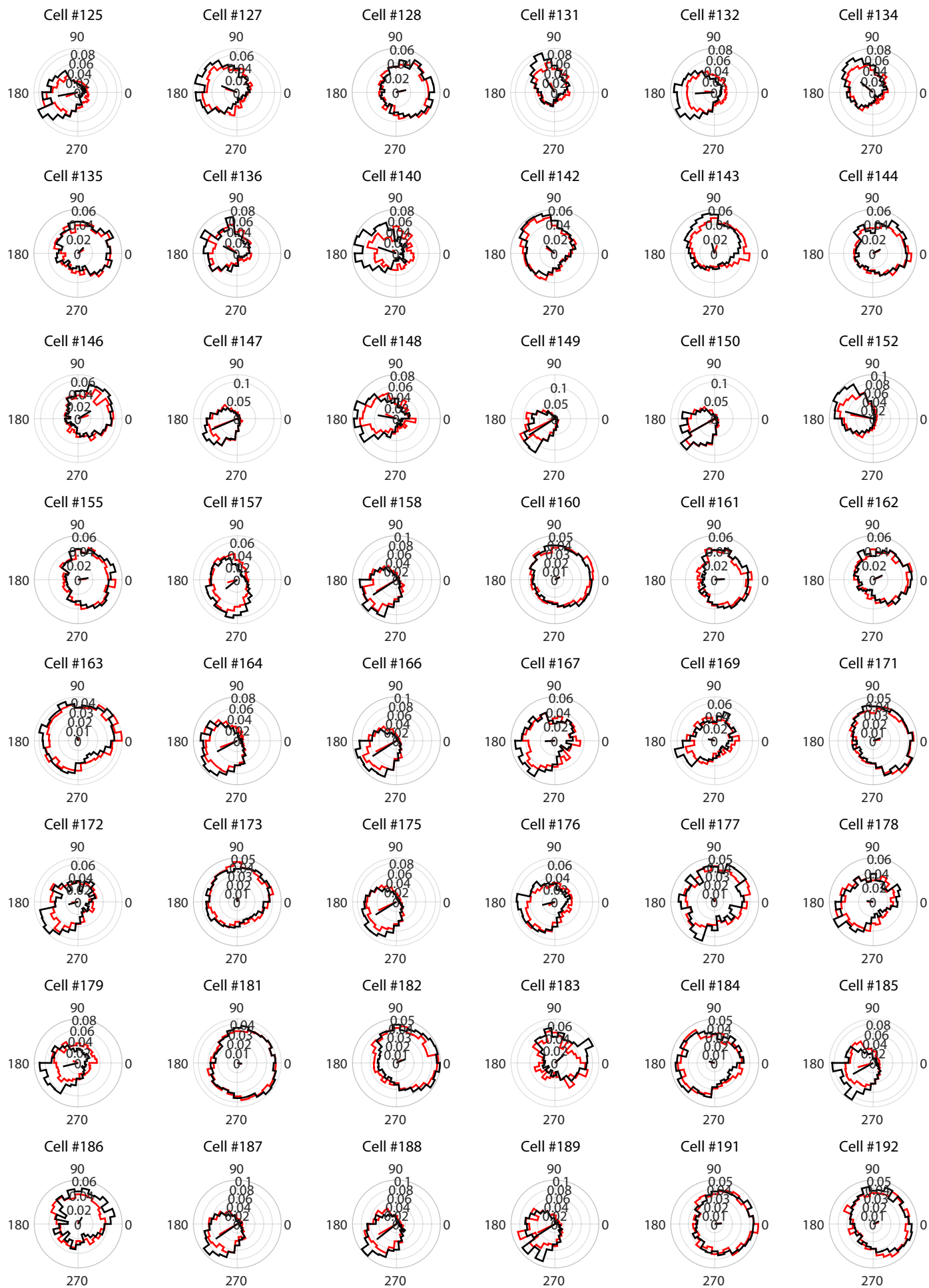

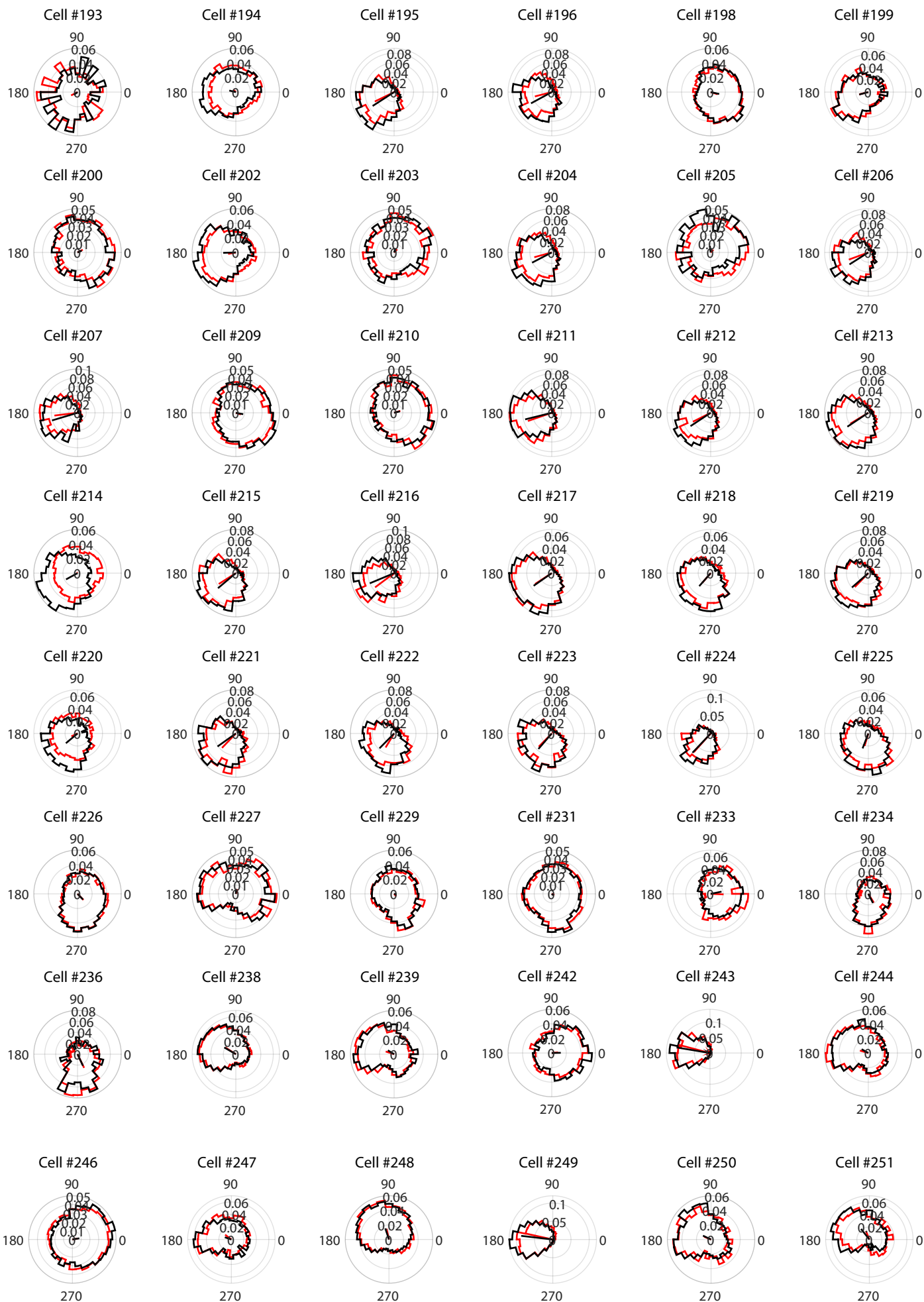

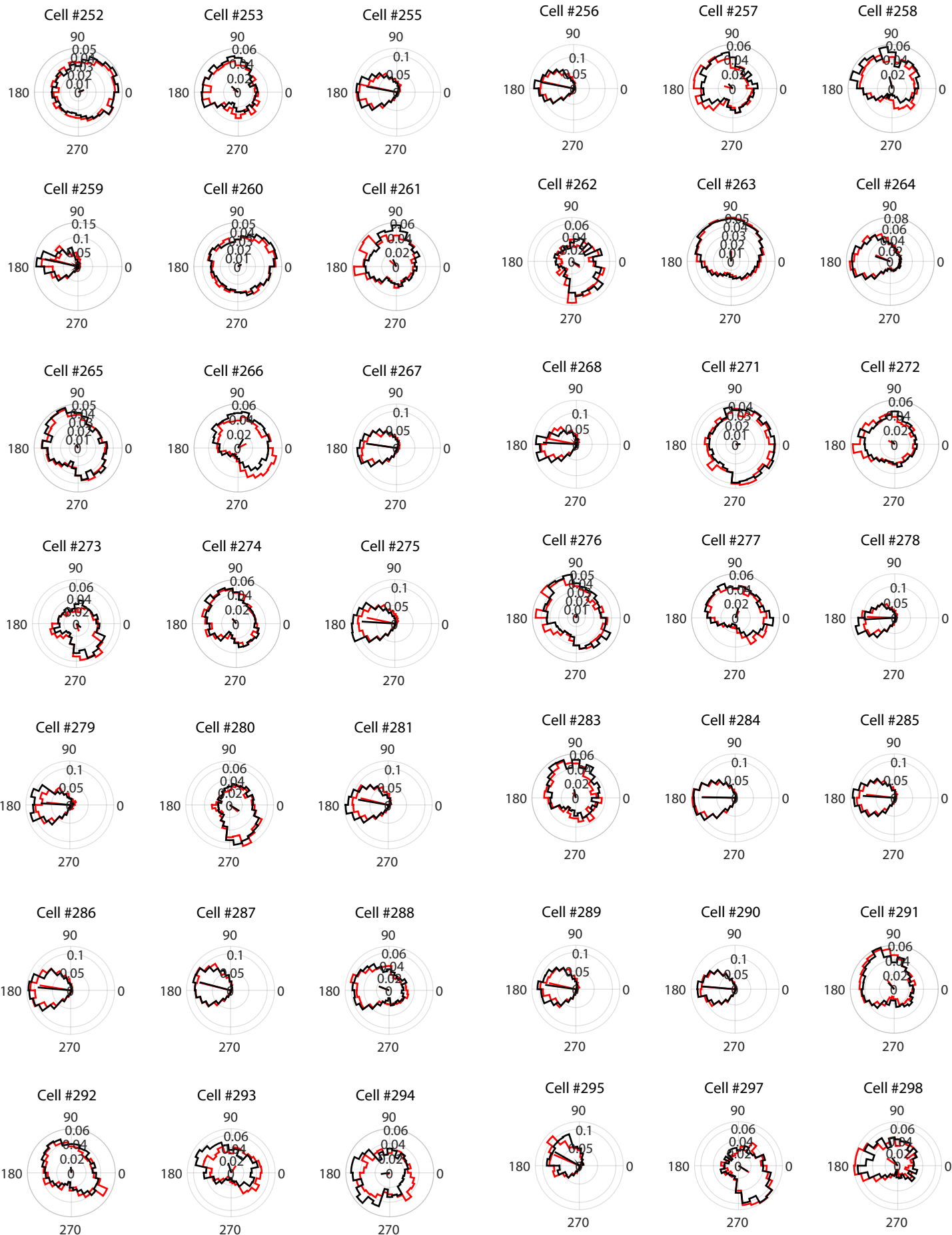

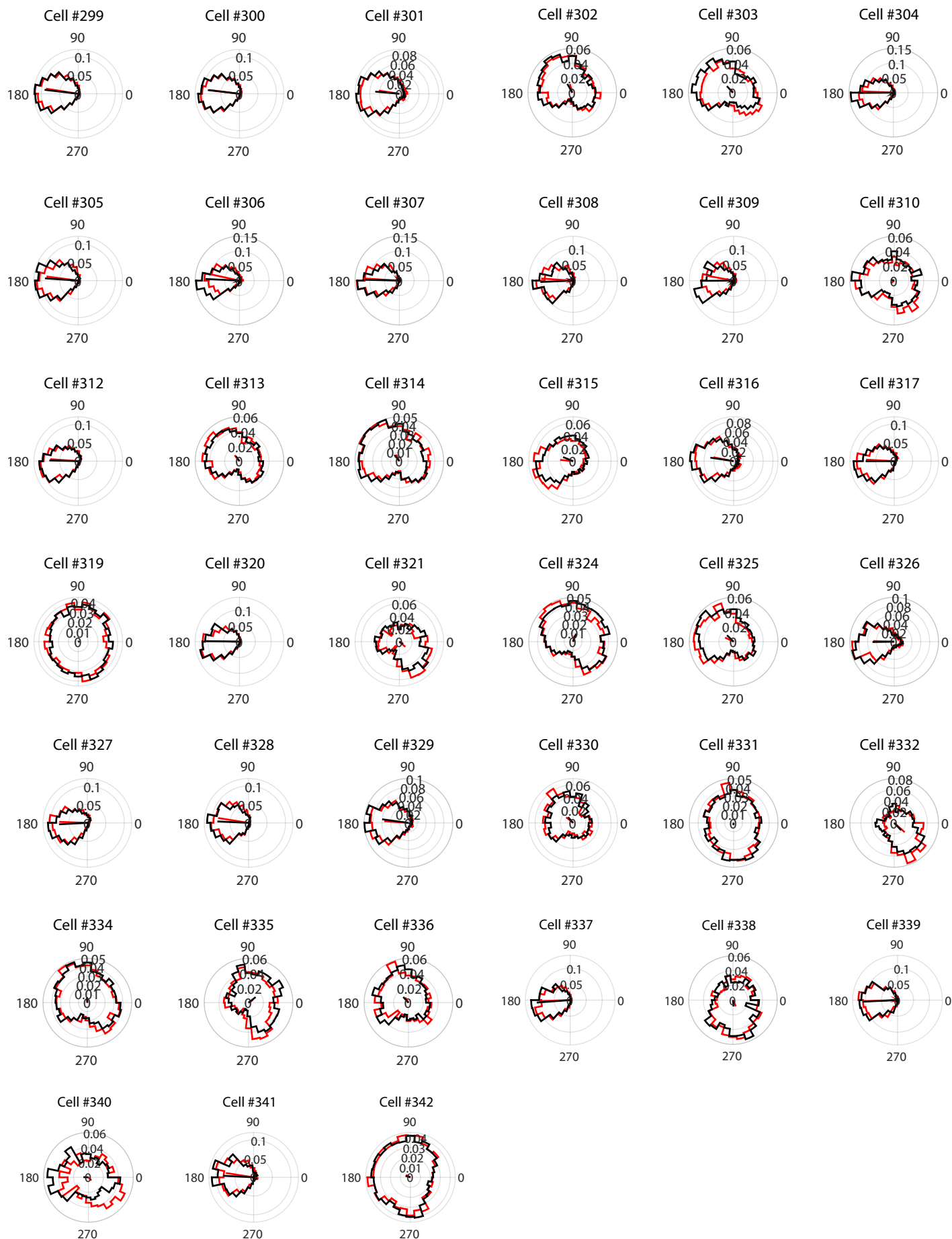

Supplement: Figure 3—source data 1. [file elife-62500-fig3-data1.pdf]

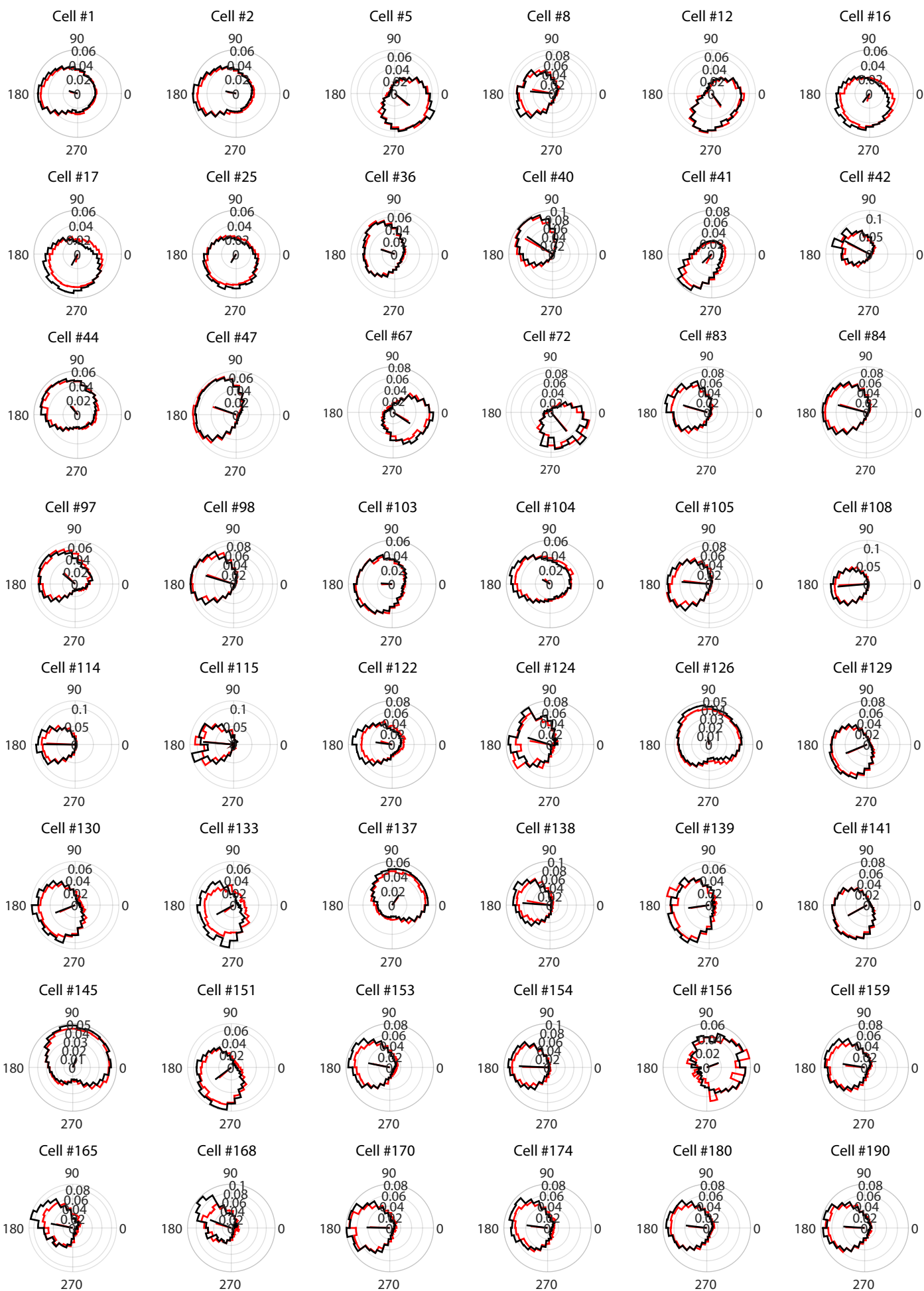

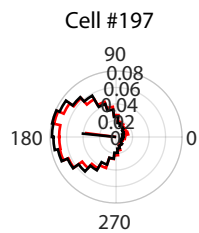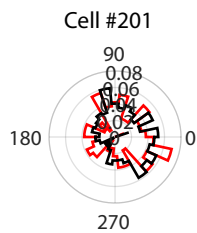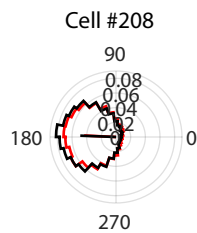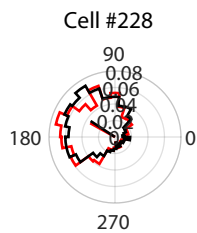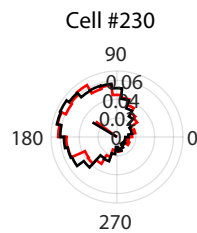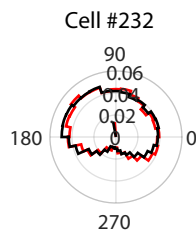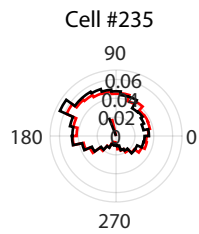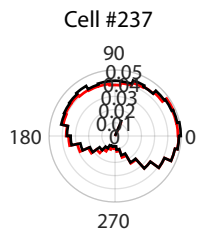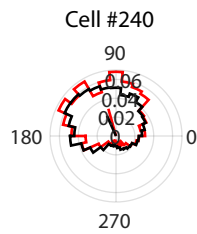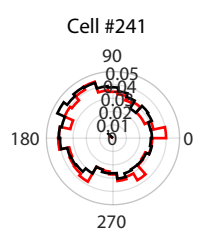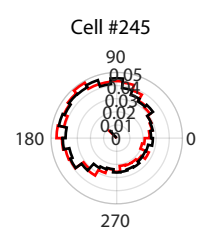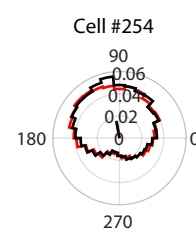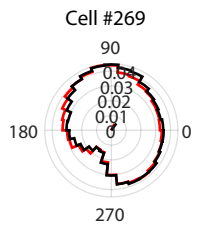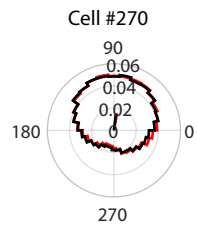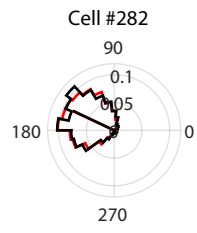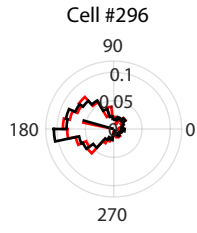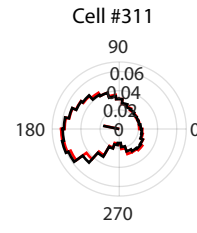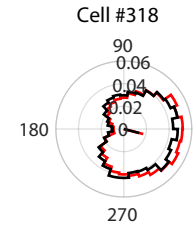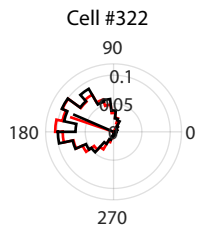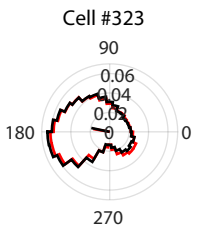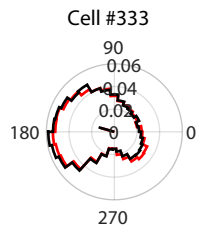

Supplement: Figure 3—source data 2. [file elife-62500-fig3-data2.pdf]

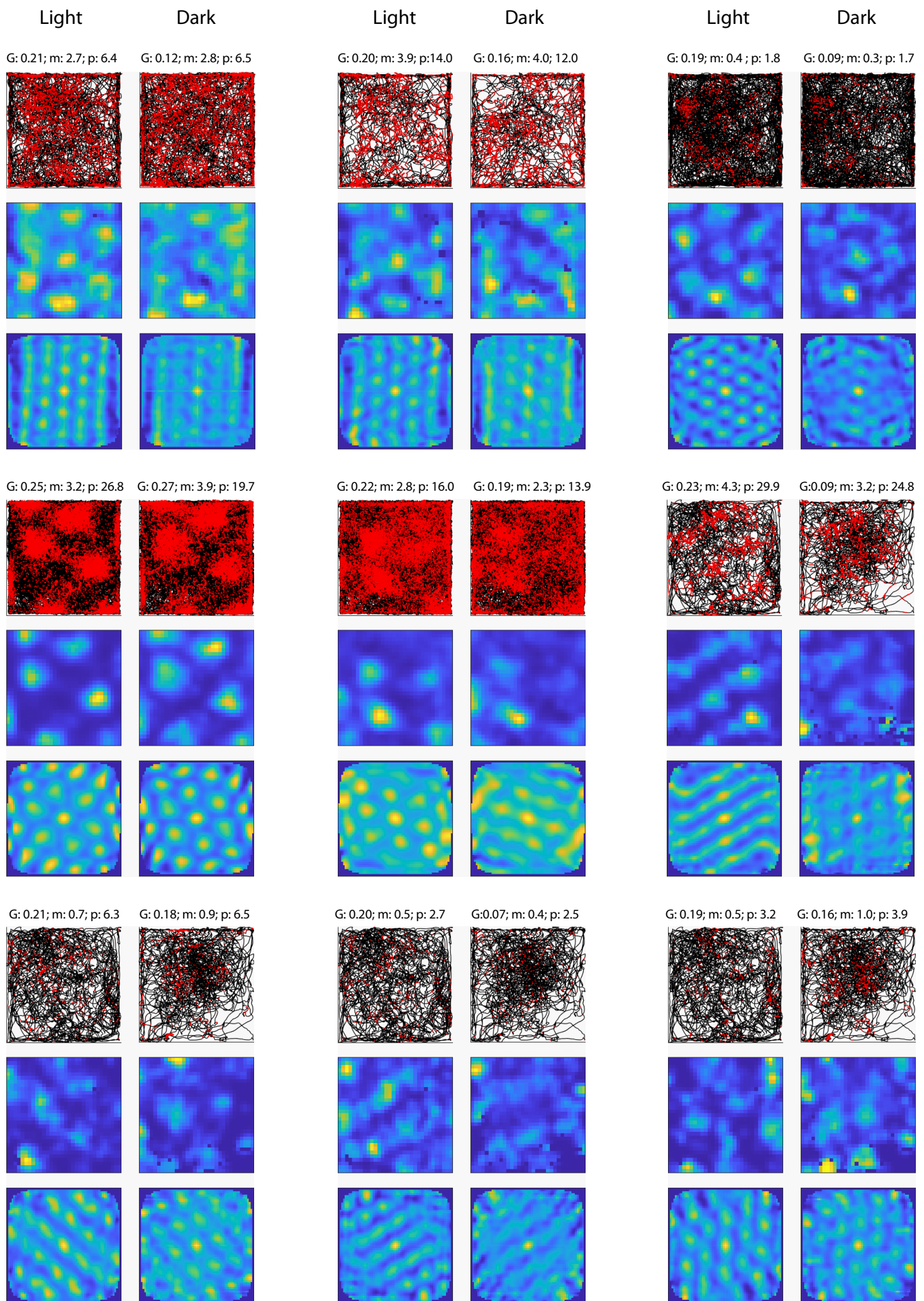



Dark

G: 0.23; m: 1.8; p: 12.3

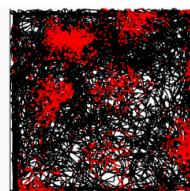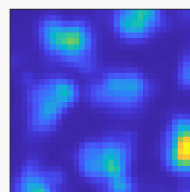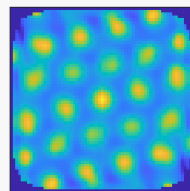

G: 0.19; m: 2.0; p: 13.2

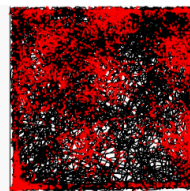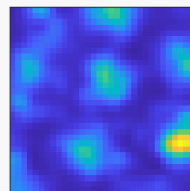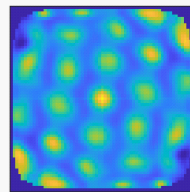

G: 0.28; m: 2.2; p: 10.0

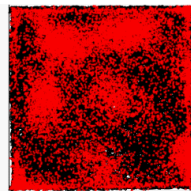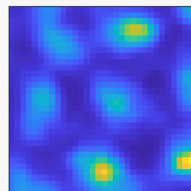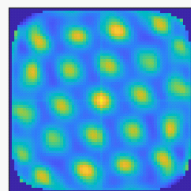

Light

Dark

G: 0.29; m: 2.3; p: 11.0

G: 0.22; m: 2.7; p: 15.8

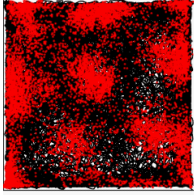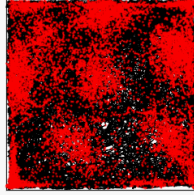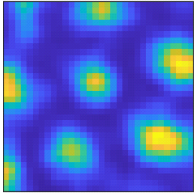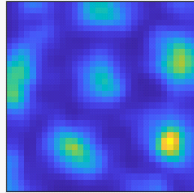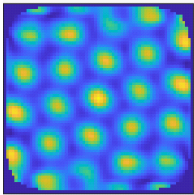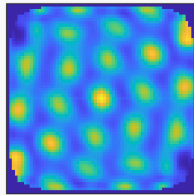

G = Grid score

m = mean firing rate

p = peak firing rate

Supplement: Figure 6—source data 1. — G = grid score; m = mean firing rate across bins of firing rate map; p=peak firing rate in firing rate map. [file elife-62500-fig6-data1.pdf]
