# Supplementary material for: Effects of visual inputs on neural dynamics for coding of location and running speed in medial entorhinal cortex
Source: eLife. 2020 Dec 10;9:e62500. doi: 10.7554/eLife.62500 (PMC7773338; doi:10.7554/eLife.62500)
Supplement: Transparent reporting form [file elife-62500-transrepform.docx]

***eLife’s* transparent reporting form**

We encourage authors to provide detailed information *within their submission* to facilitate the interpretation and replication of experiments. Authors can upload supporting documentation to indicate the use of appropriate reporting guidelines for health-related research (see [EQUATOR Network](http://www.equator-network.org/%20)), life science research (see the [BioSharing Information Resource](https://biosharing.org/)), or the [ARRIVE guidelines](http://www.plosbiology.org/article/info:doi/10.1371/journal.pbio.1000412) for reporting work involving animal research. Where applicable, authors should refer to any relevant reporting standards documents in this form.

If you have any questions, please consult our Journal Policies and/or contact us: [editorial@elifesciences.org](mailto:editorial@elifesciences.org).

**Sample-size estimation**

- You should state whether an appropriate sample size was computed when the study was being designed
- You should state the statistical method of sample size computation and any required assumptions
- If no explicit power analysis was used, you should describe how you decided what sample (replicate) size (number) to use

Please outline where this information can be found within the submission (e.g., sections or figure legends), or explain why this information doesn’t apply to your submission:

Sample sizes are reported in the Results section and Figures and/or Figure legends. The number of animals used for data collection was estimated based on previous studies using local field potential and single unit recordings in freely moving mice from our lab and in the literature. The required number of animals is largely determined by the number of grid cells present in the recordings. Based on previous studies comparing grid cells between experimental conditions, we aimed at recording 20-25 grid cells from at least 3 different mice (Fig. 6). The analysis of grid cell firing properties requires sufficient coverage of the open field environment by random exploration, usually 10-20 min. We therefore designed a typical experimental recording session to have a length of 42min so that light and dark conditions were ~20min long. To analyze the time courses of neural dynamics, we further designed experiments to contain 2-3 min alternating light and dark epochs to obtain sufficient data on transitions from light to dark or from dark to light conditions. For the analysis of the time course of grid cell spatial stability (Fig. 8), we only included grid cells recorded over multiple (five to seven) recording sessions, which is equivalent to at least 6 min random exploration time per analyzed block of activity.

**Replicates**

- You should report how often each experiment was performed
- You should include a definition of biological versus technical replication
- The data obtained should be provided and sufficient information should be provided to indicate the number of independent biological and/or technical replicates
- If you encountered any outliers, you should describe how these were handled
- Criteria for exclusion/inclusion of data should be clearly stated
- High-throughput sequence data should be uploaded before submission, with a private link for reviewers provided (these are available from both GEO and ArrayExpress)

Please outline where this information can be found within the submission (e.g., sections or figure legends), or explain why this information doesn’t apply to your submission:

The number of biological replications (number of animals, recording sessions, and number of neurons) are reported in the Results and/or Materials and Methods sections. We did not remove any outliers from the analysis. Inclusion and exclusion criteria for each analysis are described in detail in the Materials and Methods section.

**Statistical reporting**

- Statistical analysis methods should be described and justified
- Raw data should be presented in figures whenever informative to do so (typically when N per group is less than 10)
- For each experiment, you should identify the statistical tests used, exact values of N, definitions of center, methods of multiple test correction, and dispersion and precision measures (e.g., mean, median, SD, SEM, confidence intervals; and, for the major substantive results, a measure of effect size (e.g., Pearson's r, Cohen's d)
- Report exact p-values wherever possible alongside the summary statistics and 95% confidence intervals. These should be reported for all key questions and not only when the p-value is less than 0.05.

Please outline where this information can be found within the submission (e.g., sections or figure legends), or explain why this information doesn’t apply to your submission:

The results of statistical hypothesis testing (test-statistics and p-values) and measures of range of error (standard deviation, standard error of the mean, or 95% confidence intervals) are reported in the Results section and/or in Figure legends.

(For large datasets, or papers with a very large number of statistical tests, you may upload a single table file with tests, Ns, etc., with reference to sections in the manuscript.)

**Group allocation**

- Indicate how samples were allocated into experimental groups (in the case of clinical studies, please specify allocation to treatment method); if randomization was used, please also state if restricted randomization was applied
- Indicate if masking was used during group allocation, data collection and/or data analysis

Please outline where this information can be found within the submission (e.g., sections or figure legends), or explain why this information doesn’t apply to your submission:

Our analyses use within-subject comparisons.

**Additional data files (“source data”)**

- We encourage you to upload relevant additional data files, such as numerical data that are represented as a graph in a figure, or as a summary table
- Where provided, these should be in the most useful format, and they can be uploaded as “Source data” files linked to a main figure or table
- Include model definition files including the full list of parameters used
- Include code used for data analysis (e.g., R, MatLab)
- Avoid stating that data files are “available upon request”

Please indicate the figures or tables for which source data files have been provided:

Matlab code related to Fig. 2 and Fig. 4 is available on GitHub (https://github.com/hasselmonians/mle_rhythmicity; https://github.com/hasselmonians/light-modulation.)
